# Percutaneous left renal vein stenting in posterior nutcracker syndrome: technical and antithrombotic nuances – a case report

**DOI:** 10.3389/fcvm.2026.1915089

**Published:** 2026-08-25

**Authors:** Ioana-Bristena Mardare, Horia Roşianu, Adela Mihaela Șerban, Alexandra Dădârlat-Pop, Teodor Buliga, Alexandru Achim

**Affiliations:** 1 Cardiology Department, “Niculae Stăncioiu” Heart Institute, Cluj-Napoca, Romania; 2 Department of Internal Medicine, Faculty of Medicine, “Iuliu Hațieganu” University of Medicine and Pharmacy, Cluj-Napoca, Romania

**Keywords:** left renal vein compression, left renal vein stenting, posterior nutcracker syndrome, renal venous hypertension, venous stent antithrombotic management

## Abstract

**Background:**

Posterior Nutcracker Syndrome (PNCS) is a rare variant of left renal vein (LRV) entrapment in which the compression occurs between the abdominal aorta and vertebral column. Endovascular stenting may be considered in selected patients, but technical durability and antithrombotic management remain challenging.

**Case summary:**

A 64-year-old woman with chronic kidney disease and Child-Pugh A cirrhosis with portal hypertensive lesions was referred for percutaneous treatment of symptomatic PNCS. She had recurrent haematuria, left flank pain and recurrent lower urinary tract infections. Computed tomography (CT) demonstrated retroaortic LRV compression with a pre-stenotic-to-compressed segment diameter ratio of approximately 5:1 and collateral venous drainage. Dynamic renal scintigraphy showed reduced left renal uptake and invasive assessment demonstrated a 4 mmHg trans-stenotic gradient. A dedicated venous stent was deployed at the ostial LRV stenosis with limited protrusion into the inferior vena cava, achieving good expansion and no recoil. Post-procedural evolution was notable for left femoral venous access-site bleeding controlled with compression, while the LRV stent remained patent. In view of the newly implanted renal venous stent, cirrhosis-related haemostatic fragility, and early access-site bleeding, an individualized antithrombotic regimen was selected to balance stent thrombosis prevention against haemorrhagic risk, while acknowledging the current lack of standardized protocols for renal vein stenting.

**Discussion & conclusion:**

This case illustrates the importance of tailoring percutaneous treatment of PNCS to both anatomy and bleeding risk. In this patient, retroaortic ostial anatomy guided the stent deployment strategy, while early access-site bleeding in the setting of cirrhosis prompted individualized antithrombotic management.

## Introduction

Nutcracker Syndrome is the clinical term attributed to extrinsic compression of the left renal vein (LRV), most often between the superior mesenteric artery and the abdominal aorta, resulting in symptomatic renal venous hypertension marked by flank pain, recurrent microscopic or gross haematuria, pelvic venous congestion and impaired renal function. Posterior Nutcracker Syndrome (PNCS) describes a rare anatomic variant in which the LRV has a retroaortic or circumaortic course resulting in a fixed compression between the vertebral column and the abdominal aorta (1).

In contrast to renal arterial ischaemia, the clinical and functional consequences seen in PNCS are the result of impaired venous drainage and renal venous hypertension (2, 3). Therefore, the relationship between symptoms and venous entrapment should be supported by objective anatomical and haemodynamic evidence. Although most proposed thresholds derive from experience with Anterior Nutcracker Syndrome, findings such as marked narrowing of the compressed LRV segment, a pre-stenotic-to-compressed segment diameter ratio >4–5, collateral venous drainage and/or a renocaval pressure gradient ≥3 mmHg may help support the haemodynamic relevance of posterior LRV compression (3, 4).

Treatment is individualized according to patient-specific clinical, anatomical and functional considerations and it can range from conservative management in mildly symptomatic patients, to decompression techniques in cases of persistent or severe symptoms with progression to renal functional impairment (2, 3). Surgical options include LRV transposition, renal autotransplantation and, less commonly, venous bypass procedures, whereas endovascular LRV stenting has emerged as a less invasive alternative, which may therefore be better suited to older or more comorbid patients (2, 5, 6). LRV stenting, however, presents specific technical and post-procedural challenges. Adequate stent sizing, radial force, ostial coverage and anchoring are essential to prevent persistent stenosis, restenosis, thrombosis or migration (6–10). Published series report favourable results in selected patients, but PNCS-specific management evidence remains limited and post-stenting antithrombotic strategies vary considerably (6, 9, 11). These uncertainties are amplified in patients with cirrhosis, in whom haemostatic alterations create a fragile balance between bleeding and thrombotic risk.

**Figure 1:**
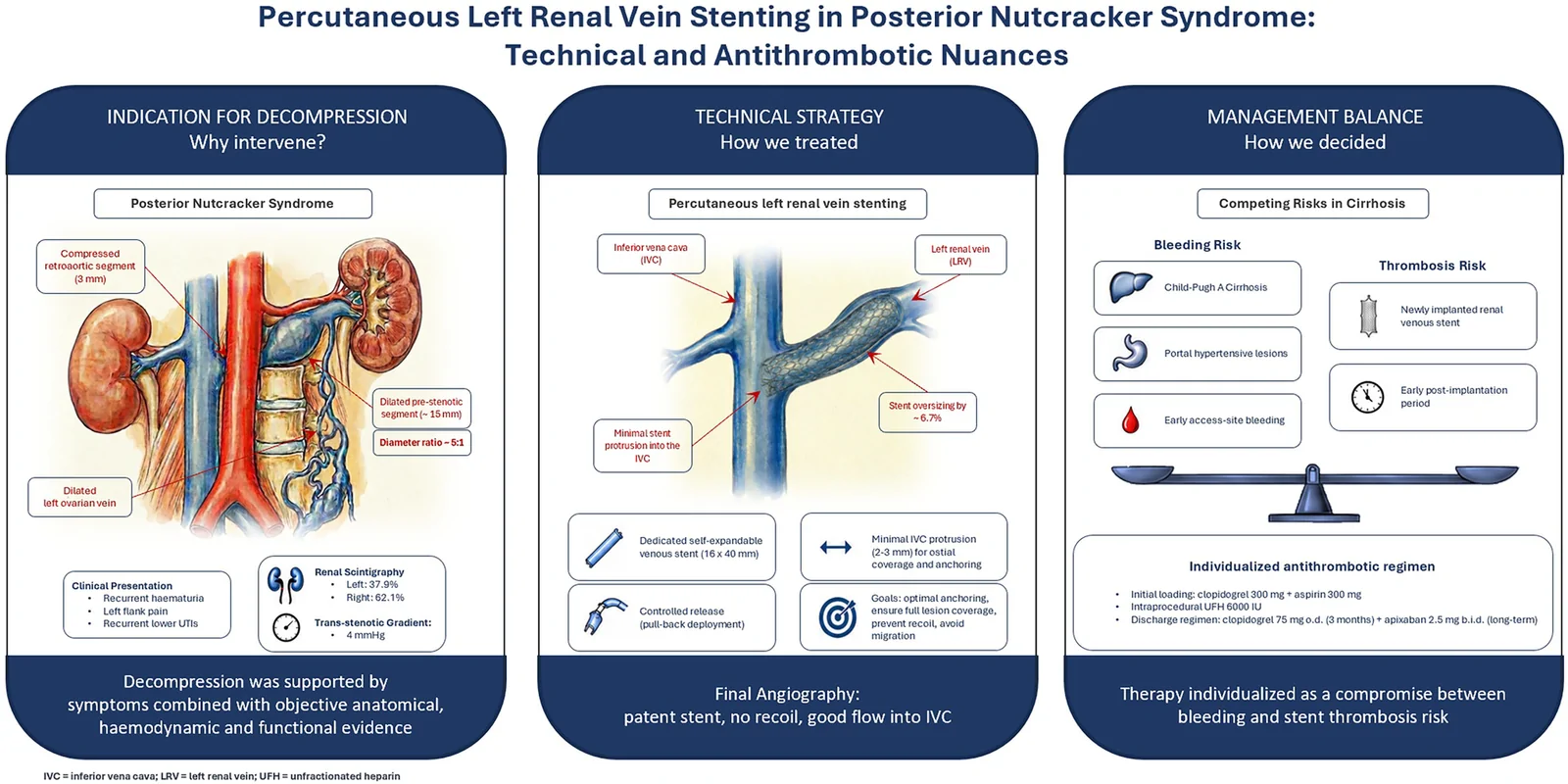
Central illustration. Percutaneous Left Renal Vein Stenting in Posterior Nutcracker Syndrome: Technical and Antithrombotic Nuances. Case summary illustrating the diagnostic rationale, technical strategy, and antithrombotic decision-making in a patient undergoing percutaneous left renal vein stenting for posterior Nutcracker syndrome.

We herein describe a case of percutaneous LRV stenting for PNCS in a 64-year-old patient with Child-Pugh A cirrhosis and portal hypertensive lesions, complicated by early access-site bleeding and requiring individualized antithrombotic management (Figure 1).

## Case presentation

A 64-year-old woman with known chronic kidney disease stage KDIGO G3a and compensated alcohol-related liver cirrhosis was electively admitted for percutaneous treatment of PNCS.

Chronic kidney disease had been diagnosed approximately 2 years earlier and was already under nephrological surveillance, although its aetiology had not been definitively established.

Her liver disease was classified as Child-Pugh A and was complicated by grade I oesophageal varices, portal hypertensive gastropathy and chronic left portal vein thrombosis. She was receiving primary prophylaxis with a non-selective beta-blocker.

The diagnosis of PNCS had been established before referral following evaluation for episodic left flank pain that had been present for several years and had worsened during the preceding year, recurrent macroscopic haematuria for approximately 6 months, and culture-confirmed lower urinary tract infections occurring approximately every 2 months. The flank pain and haematuria persisted between infectious episodes and despite antibiotic treatment, suggesting that recurrent infection alone did not fully account for the clinical presentation.

**Figure 2:**
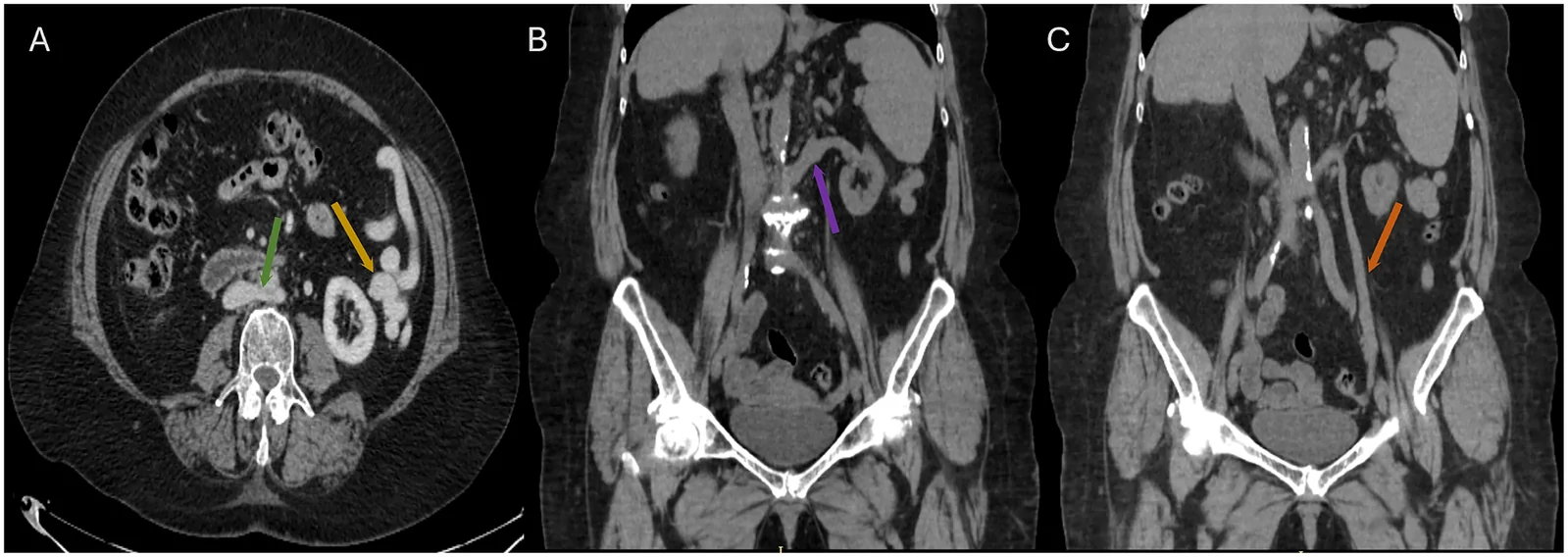
Diagnostic CT findings. **(A)** Axial venous-phase CT showing (1) marked compression of the retroaortic LRV between the abdominal aorta and vertebral body, with a minimal diameter of approximately 3 mm (green arrow) and (2) collateral venous drainage through splenorenal pathways (yellow arrow). **(B)** Pre-stenotic dilatation of the LRV measuring approximately 15 mm (purple arrow), yielding a pre-stenotic-to-compressed segment diameter ratio of approximately 5:1. **(C)** Descending retroperitoneal pararenal collateral drainage toward the left gonadal vein (orange arrow).

Abdominal CT demonstrated a retroaortic LRV entrapped between the abdominal aorta and the vertebral body. The compressed segment measured 3 mm in anteroposterior diameter, whereas the pre-stenotic segment measured approximately 15 mm, yielding a pre-stenotic-to-compressed segment diameter ratio of approximately 5:1 (Figures 2A,B). The examination excluded urinary tract calculi, hydronephrosis, collecting-system filling defects and bladder wall abnormalities, while also demonstrating important spleno-renal collateral circulation and a descending retroperitoneal pararenal venous pathway draining into the left gonadal vein (Figure 2C), supporting the haemodynamic relevance of the venous compression.

Dynamic renal scintigraphy with 99mTc-MAG3 (100 MBq) demonstrated reduced left renal tracer uptake and a lower-amplitude left renographic curve, with preserved functional phases and no pyelocaliceal dilatation or obstructive drainage pattern; diuretic administration was not required. Split renal function was 37.9% on the left and 62.1% on the right.

In the absence of a more plausible explanation for the disproportionate reduction in left renal function, the combination of persistent symptoms, marked retroaortic LRV compression with collateral venous drainage, and a 4 mmHg trans-stenotic gradient supported the interpretation that PNCS was contributing to the patient's renal dysfunction. On this basis, the indication for LRV decompression was established. Surgical LRV transposition and percutaneous venous stent implantation were discussed as therapeutic options. Following multidisciplinary assessment and shared decision-making, an endovascular strategy was chosen in view of the patient's age, comorbid profile, operative risk and favourable focal anatomy for percutaneous treatment.

On admission, the patient was haemodynamically stable and physical examination was notable only for left flank percussion tenderness. Of note, 2 weeks before admission, she had been diagnosed with a culture-confirmed *Escherichia coli* urinary tract infection and had completed antibiotic treatment. At admission, she was afebrile, without leukocytosis or a relevant inflammatory response; urinalysis showed no evidence of active urinary infection, and urine culture was negative.

Prior renal ultrasonography showed mildly increased cortical echogenicity with preserved parenchymal thickness of approximately 1.3–1.5 cm, while Uro-CT measured the right and left kidneys at approximately 9.0 and 9.8 cm, respectively.

Laboratory testing showed moderate renal dysfunction, with serum creatinine of 1.17 mg/dL and an estimated glomerular filtration rate of 49.27 mL/min/1.73 m^2^. Available previous measurements showed relatively stable KDIGO stage G3a renal function, with serum creatinine ranging from 1.07 to 1.38 mg/dL and estimated glomerular filtration rates from 43 to 54 mL/min/1.73 m^2^. Previous urinary assessment showed mild proteinuria without relevant albuminuria. Baseline haemoglobin was 14.8 g/dL, platelet count was mildly reduced at 139 × 10^3^/µL, and INR was 1.16. Liver synthetic function was preserved, with total serum protein and albumin within normal limits at 6.4 g/dL and 3.9 g/dL, respectively.

After preprocedural loading with aspirin 300 mg and clopidogrel 300 mg, selective left renal venography was performed through dual femoral venous access. A 10 Fr right femoral venous sheath was used for stent delivery and was pre-closed with one Perclose ProGlide™ system (Abbott Vascular, Santa Clara, CA, USA). A 6 Fr left femoral venous sheath was used for contralateral contrast injections during stent positioning, avoiding contrast injection through the delivery sheath or further upsizing of the right femoral access.

Selective cannulation of the LRV was performed via a 5 Fr JR diagnostic catheter. Contrast injection demonstrated ostial stenosis of the LRV at its drainage into the inferior vena cava, with a trans-stenotic gradient of 4 mmHg.

An ultra-stiff 0.035-inch guidewire was advanced distally into the renal hilum. Stent positioning was guided by contralateral contrast injections.

**Figure 3:**
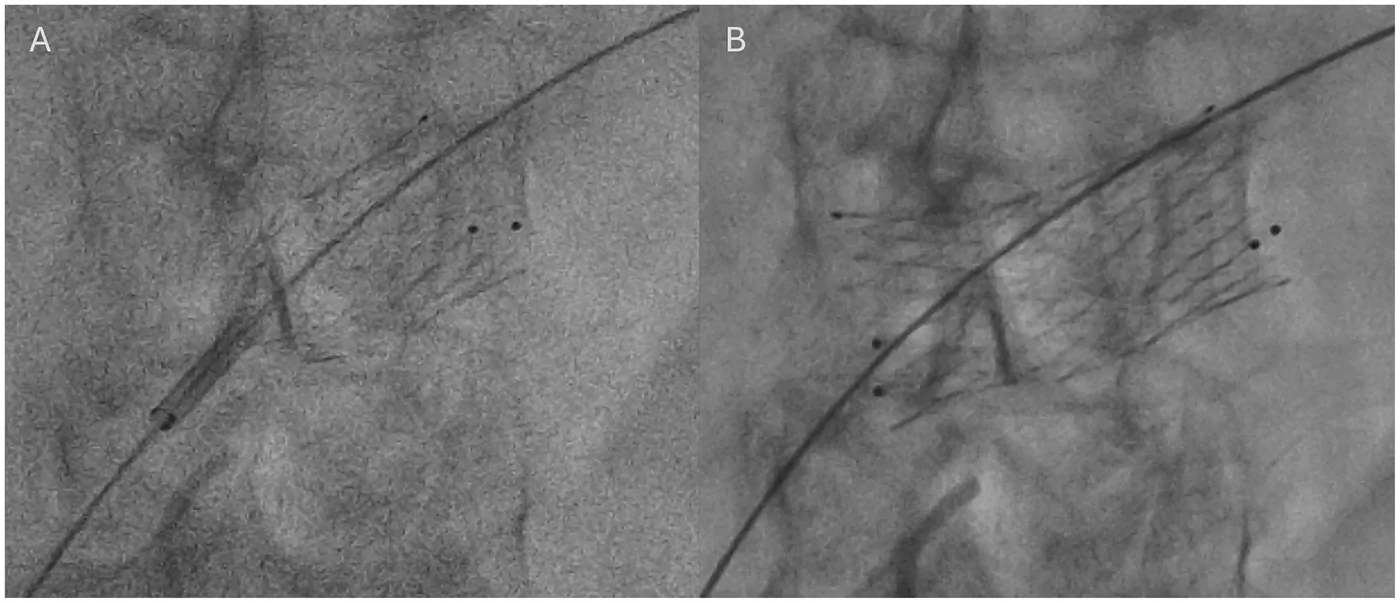
**(A)** Stent deployment across the ostial retroaortic left renal vein stenosis. **(B)** Final angiographic result showing a patent, well-expanded stent with preserved flow into the inferior vena cava and no recoil.

A 16 × 40 mm VENOVO venous stent was selected relative to the approximately 15-mm maximum pre-stenotic LRV diameter, corresponding to approximately 6.7% diameter oversizing. More aggressive oversizing was considered unnecessary because the severe focal 3-mm narrowing provided an additional anchoring zone. Final deployment was performed using controlled pull-back release with intentional 2–3 mm protrusion into the inferior vena cava to maintain ostial positioning and avoid distal stent displacement toward the renal hilum during self-expansion (Figure 3A).

Final angiography demonstrated a patent, well-expanded stent with relief of the ostial stenosis and no residual recoil (Figure 3B). Intraprocedural anticoagulation consisted of 6,000 IU unfractionated heparin. No immediate intraprocedural complications were recorded.

Both femoral venous sheaths were removed immediately after completion of the procedure. Haemostasis of the 10-Fr right femoral access was achieved using the previously placed Perclose ProGlide™ device, whereas the 6-Fr left femoral access was managed by manual compression until haemostasis, followed by application of a compressive dressing intended to remain in place for 4 h.

**Figure 4:**
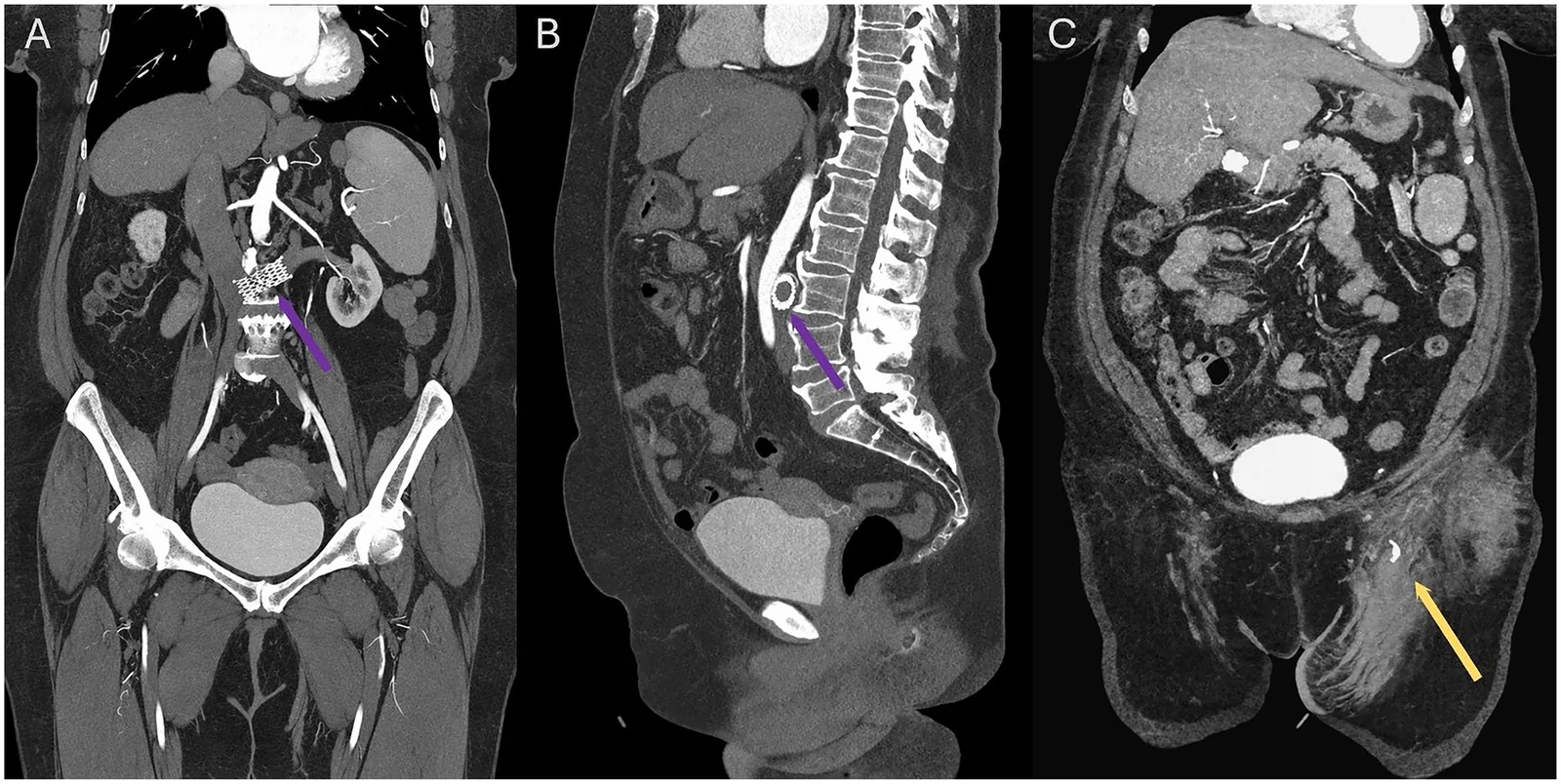
Postprocedural contrast-enhanced CT scan. **(A)** and **(B)** Patent left renal vein stent (purple arrows). **(C)** Active bleeding from the left femoral vein access site with associated proximal thigh haematoma (yellow arrow).

Approximately 2 h after the procedure, severe pain arose in the left groin and proximal thigh and radiated toward the flank. Urgent venous-phase CT angiography demonstrated active bleeding from the left femoral access site beneath the dressing, with proximal thigh soft-tissue infiltration (Figure 4C). No retroperitoneal, intra-abdominal, pelvic or perirenal bleeding, renal vein injury or renal infarction was identified. The LRV stent remained patent and bilateral renal enhancement and excretory function were preserved (Figures 4A,B). Anticoagulation was therefore withheld that evening.

The compressive dressing was reinforced, with prompt pain improvement. Haemoglobin was 13.8 g/dL versus 14.8 g/dL at baseline, platelet count 122 × 10^3^/µL, INR 1.32 and activated partial thromboplastin time 51.4 s. Doppler ultrasound showed a superficial 41 × 16 mm organized haematoma without active bleeding or pseudoaneurysm. No transfusion or reintervention was required, and apixaban 2.5 mg twice daily was introduced the following day after bleeding had ceased.

The patient was discharged on day 3 after stent implantation in good clinical condition, asymptomatic and haemodynamically stable. The discharge antithrombotic regimen consisted of clopidogrel 75 mg once daily for 3 months and apixaban 2.5 mg twice daily long term, together with proton-pump inhibitor therapy and continuation of gastroenterological management.

At 1-month follow-up, the patient reported complete resolution of flank pain and haematuria. Doppler ultrasound demonstrated preserved phasic/modulated venous flow within the stented LRV, supporting stent patency.

## Discussion

PNCS remains an uncommon and anatomically distinct form of LRV entrapment and its optimal interventional management is not standardized. Reported strategies reflect this uncertainty, ranging from open LRV transposition to hybrid reconstruction, extravascular and endovascular stenting. Rodríguez-Morata et al. described two purely endovascular cases treated with gonadal vein embolization, angioplasty and retroaortic LRV stenting (12). Stawiarski et al. reported a hybrid approach combining open LRV transposition with endovascular stenting, including surgical fixation of the stent to reduce migration risk (13). By contrast, St Hilaire et al. and Marone et al. described open surgical transposition alone, without an endovascular component (14, 15), whereas Yu et al. reported extravascular stenting as a means of avoiding intraluminal stent-related complications and the need for prolonged post-procedural antithrombotic therapy (16).

The present case adds to the limited PNCS literature by illustrating a purely percutaneous strategy in an older, higher-risk patient in whom anatomical, technical and haemostatic factors all shaped management.

LRV stenting has shown encouraging mid-term results, but available data remain limited and are derived mainly from heterogeneous, non-posterior-specific series. In a 2025 systematic review, Sarikaya et al. identified 170 patients treated with endovascular stenting, with reported symptom resolution in 76% and a reintervention rate of 11.3%, while emphasizing the limitations imposed by small cohorts, variable follow-up, and inconsistent outcome reporting (17). Avgerinos et al. reported 2-year primary patency of 85.2% and primary-assisted patency of 100%, although 3 of 18 patients required reintervention for recurrent symptoms or restenosis (6). More recently, Anan et al., in a larger long-term series from the same group, reported complete or partial symptom improvement in 90.7% of patients, with primary patency rates of 89.9% at 1 year and 82.8% at 4 years and primary-assisted patency of 100% (18).

Nevertheless, stent-related safety concerns remain relevant. Migration is among the most important complications, reported in approximately 4.9%–6.7% of cases, with displacement described into the inferior vena cava, right atrium, right ventricle, or hilar LRV (8, 11).

Other reported complications include restenosis, recurrent symptoms, stent kinking and flow-limiting inferior vena cava obstruction related to suboptimal stent geometry or excessive oversizing (6, 7).

However, posterior-specific long-term complication rates are not well defined and the fixed aorto-vertebral compression may theoretically add concern for persistent external forces acting on the implanted stent.

Oversizing by approximately 20% relative to the maximum pre-stenotic LRV diameter has been described as a strategy to improve wall apposition, radial stability and anchoring in a venous segment characterized by marked calibre mismatch, thereby reducing migration risk (7, 18).

Radial force is also important, as these stents usually cannot be post-dilated, so the compromise of using other types of stents (i.e., balloon expandable stents) is not recommended. After release, device nose-cone must be centralized and withdrawal done slowly, similar to the approach seen in transcatheter aortic valve implantation (TAVI) procedures (7). The use of a contralateral “secondary venous access” for accurate inferior vena cava angiography is also inspired by techniques routinely employed in the TAVI field.

Partial extension into the inferior vena cava may be used as a strategy to improve ostial coverage and reduce migration risk. However, this creates a technical trade-off: protrusion should be sufficient to secure the ostium, but limited enough to avoid converting the renal vein stent into a flow-limiting or obstructive structure within the inferior vena cava, which may also complicate future venous access or reintervention (6, 7).

These published concerns directly informed our procedural strategy. Given the junctional location of the lesion, the aim was to restore renal vein outflow while avoiding either incomplete ostial treatment or excessive caval extension. Although oversizing by approximately 20% has been described, a more limited degree of 6.7% was selected in this case because the severely compressed 3-mm ostial segment was expected to provide an additional anchoring zone, reducing the perceived need for more aggressive oversizing of the dilated pre-stenotic LRV. A dedicated self-expandable venous stent was therefore selected to provide radial support, with limited oversizing and controlled pull-back deployment used to maintain stable positioning and prevent distal displacement toward the renal hilum. Final angiography confirmed stent patency, absence of recoil and preserved drainage into the inferior vena cava. The authors acknowledge that final stent deployment can be relatively abrupt; therefore, particular care should be taken to ensure slow and controlled release, with continuous adjustment of the push–pull forces applied to the delivery catheter throughout the deployment process.

Antithrombotic therapy after LRV stenting for Nutcracker Syndrome remains poorly standardized, with published reports using heterogeneous regimens. Chen et al. used low-molecular-weight heparin for 3 days combined with clopidogrel for 30 days and aspirin for at least 3 months (11); Wang et al. used warfarin for 6–12 months (9); and Avgerinos et al. preferred dual antiplatelet therapy for 1–3 months followed by lifelong low-dose aspirin (6). Broader venous-stent consensus recommendations, derived mainly from iliofemoral and iliocaval interventions, generally favor anticoagulation for 6–12 months after stenting; however, these provide only indirect guidance and cannot be directly extrapolated to renal vein stenting (19).

Because no standardized antithrombotic protocol exists after LRV stenting, the selected regimen represented an individualized and empirical strategy. Clopidogrel was prescribed for 3 months to provide antiplatelet coverage during the early post-implantation period, while long-term apixaban 2.5 mg twice daily was intended to reduce the risk of renal vein stent thrombosis. This approach reflected the competing thrombotic and haemorrhagic risks in this patient and should not be generalized. Longer clinical and imaging follow-up is required to assess its efficacy in preventing in-stent thrombosis and restenosis.

## Conclusion

This case supports the feasibility of percutaneous LRV stenting in a selected patient with PNCS. Limited oversizing, precise ostial positioning and controlled deployment were associated with early technical success, 1-month stent patency and short-term symptom resolution. Antithrombotic therapy required an individualized, empirical approach because stent thrombosis prevention had to be balanced against cirrhosis-related haemorrhagic risk and early access-site bleeding. Longer follow-up is required to assess migration, restenosis, thrombosis, bleeding and renal functional evolution.

## Data Availability

The original contributions presented in the study are included in the article/Supplementary Material, further inquiries can be directed to the corresponding author.
